# Cultural Proficiency in First Nations Health Research: A Mixed-Methods, Cross-Cultural Evaluation of a Novel Resource

**DOI:** 10.3390/ijerph20010039

**Published:** 2022-12-20

**Authors:** Paul Saunders, Aunty Kerrie Doyle

**Affiliations:** 1 School of Medicine, Western Sydney University, Campbelltown, NSW 2560, Australia; 2 Translational Health Research Institute, Western Sydney University, Campbelltown, NSW 2560, Australia

**Keywords:** cultural competency, Aboriginal Australians, First Nations health, First Nations research, cross-cultural comparison

## Abstract

Recent efforts have illustrated the efficacy of culturally proficient approaches to research, underpinned by robust partnerships between researchers and First Nations peoples and communities. This article seeks to determine differences in approaches to First Nations research engagement perceptions between First Nations and non-First Nations researchers, as well as whether participation in a cultural proficiency workshop improved the perceived cultural proficiency of non-First Nations health researchers. Also, whether a set of novel cultural proficiency resources, designed in the Sydney region could be applied broadly across First Nations contexts within Australia. The evaluation adopted a mixed-methods, cross-cultural (First Nations and non-First Nations) design to appraise the novel cultural proficiency resources, identifying participant perceptions to First Nations research engagement, as well as views regarding the feasibility of universal application of the resources. A quantitative pre- and post-workshop evaluation was also undertaken to measure differences in self-reported cultural proficiency. Qualitative data underwent thematic analysis and quantitative data were analysed applying *t*-tests. Both qualitative and quantitative evaluation showed minimal variation between the cultural groups regarding research engagement perceptions, based on viewing of the online resources. A statistically significant increase in self-reported cultural proficiency was found in non-First Nations workshop participants. Cultural proficiency education and training programs that promote an immersive, interactive, and ongoing framework can build the perceived cultural proficiency of non-First Nations health researchers, however First Nations expertise must validate this perceived cultural proficiency to be beneficial in practice. Based on the research findings, applying the underlying ethical principles of First Nations research with a local, context-centred approach allows for the broad application of cultural proficiency research education and training programs within Australia.

## 1. Introduction

First Nations people and communities of what is now referred to as, Australia, possess a rich and diverse collection of knowledges, practices, and philosophies which are underpinned by principles of respect, integrity, reciprocity, connection, and responsibility [1,2]. The term ‘First Nations’ refers collectively to the original inhabitants of Australia, also known as Aboriginal and Torres Strait Islander peoples. First Nations communities include over 250 diverse nations and language groups that have operationally, caringly, and successfully inhabited Australia for more than 65,000 years [2,3]. Despite the invasion and continued colonisation of Australia by European powers, First Nations peoples and communities have maintained robust cultural identities and ancient social systems that have ensured a continued connection to Country, Lore, and Community [4]. However, the relentless impact of colonisation continues to bring about inequalities in many facets of life for First Nations people, including health outcomes, such as higher rates of mental illness and lower life expectancy [5,6].

There has and continues to be a domination of Western research narratives and approaches on First Nations communities, adopting a deficit frame, affecting data analysis and interpretation, and translation of research findings [7,8,9]. The result of adopting such frame ‘problematises’ First Nations peoples and represents them as recipients of their own neglect, perpetuating the deficit discourse that manifests in sustained poorer health outcomes [10,11]. Research on First Nations populations in Australia has historically been associated with coercion, disenfranchisement, and injustice, resulting in unfavourable research-related outcomes in First Nations communities, in part, due to the cross-culturally naïve non-First Nations researchers [12,13]. Martin and Mirraboopa describe this research practice as ‘terra nullius research’, whereby First Nations participants are mere objects “to be seen but not asked, heard, or respected” [14] p. 203. Establishing decolonising research partnerships whereby First Nations research participants and communities are empowered to direct research, including prioritising First Nations methods of inquiry, encourages self-determining research practice and amplifies the voice of First Nations communities within the context of First Nations health research [15,16,17] pp. 115–116.

Research can provide the health sector with valuable and accurate answers to often complex medical enigmata. As Western paradigms of research have progressed over millennia, the concept of health and disease has evolved to encompass an array of social determinants impacting on and influencing health outcomes [18]. This holistic concept has always been evident in the definition of First Nations health, where health (both collectively and individually) is shaped by social, cultural, ecosystemic, and spiritual factors, focusing on much more than mere solitary biomedical problems [19].

‘Indigenist’ research considers, adopts, and applies the values of First Nations cultures in the design, conduct, analysis, interpretation, and translation of research, involving First Nations researchers and participants [7,9,16]. Rigney [7] illustrates the importance of Indigenist research as a way of liberating First Nations peoples and communities from oppressive narratives and structures that impede their right to self-determination. Key to the principles of Indigenist research, is a focus on emancipation through First Nations-led research that privileges the voice of First Nations participants, and informs a First Nations agenda, grounded principally in historical and contemporary social and political oppression. It is only through this approach that liberation of First Nations peoples and communities can progress. Indigenist research methodologies, such as, for example ‘Yarning’ and ‘Dadirri’, are characterised by centring First Nations ontologies, axiologies, and epistemologies to support and guide a goal of self-determination for First Nations peoples [20,21].

While many First Nations scholars assert the importance of Indigenist research, it is also important that non-First Nations researchers understand this significance and can effectively act as allies for the political liberation of First Nations peoples and communities, through research that centres a liberatory epistemology [7]. A lack of inclusive Indigenist conceptualisation in research practice by non-First Nations researchers has historically resulted in biased analysis and interpretation of First Nations research data by non-First Nations researchers who have not considered the sweeping socio-political impacts of colonisation, Indigenist research paradigms, the cultural diversity in and between First Nations peoples and communities, or who have primarily focused on a deficit approach to First Nations research [22,23,24,25]. The former infers freedom of choice in determining one’s health, placing the onus on the individual rather than the socio-political constructs they are so tightly bound by. Such misanalysis and misinterpretation by non-First Nations researchers present a significant problem when translating research findings to health practice, particularly when the inaccuracy occurs at a higher research level rather than at an individual health practitioner level [26]. This complexity, often incomprehensible to many non-First Nations health practitioners, frequently culminates in deficient ‘evidence-based’ care and, subsequent poor healthcare provision for First Nations people [27].

The health inequalities experienced by First Nations people and communities highlight a lack of cross-cultural capability understanding, appreciation, and application by the non-First Nations population [28]. Cross-cultural capability is an encompassing term, including models of cultural awareness, cultural competence, cultural responsiveness, cultural sensitivity, and cultural safety, that seeks to critically analyse current systems of inequity, encourage critical self-reflection, and deconstruct and reconstruct the attitudes, knowledges, and skills required to effectively interact and engage with people from other cultures in an affirming and self-determining way [29]. The various models identified within cross-cultural capability have been increasingly critiqued, with particular models demonstrating superiority, such as cultural safety [28]. Nonetheless, for the purposes of this evaluation, a focus on the cultural proficiency model was investigated, concordant with the resources under consideration. Considering the ever-apparent inequitable impacts of cultural ignorance, Cross et al. [30] explored the idea of a cultural competence continuum whereby individuals can move up and down the continuum depending on their capability when placed within a cross-cultural setting. At one end of the continuum, cultural destructiveness seeks to intentionally impair cultures, while at the opposite end, those with an informed understanding and skill set to aid in the empowerment of different cultures are considered culturally competent, with the application of this advanced knowledge and skill set, while exercising cultural humility, within a real-world setting to contribute to positive outcomes, considered culturally proficient. Cultural humility is a lifelong commitment to critical self-reflection to act on power differentials within relationships, and to develop reciprocal, affirming partnerships [31]. It is important to note that while the terms ‘competent’ and ‘proficient’ traditionally denote a degree of static mastery, within this context, it is a dynamic state of always ‘becoming’, rather than ‘being’, creating an ever-elusive goal for those undertaking such a journey [32]. 

Developing the cultural proficiency of non-First Nations health researchers would be one way to help address the inequities in First Nations-related health research [33]. It is the attitudes and behaviours of health care providers and researchers that are either culturally competent or not [34], and many non-First Nations health professionals continue to acknowledge their cultural incompetence [35]. An evidenced-based, community-informed program embedded into the training mechanisms of all health professionals to skill practitioners and researchers in cultural proficiency would decrease racism in health and research settings [36,37], by centering the value of social justice and giving voice to First Nations people and communities as custodians of their health [38]. The development of long-term, decolonising relationships with First Nations communities is key to undertaking successful research, and ultimately reducing health research disparities within First Nations communities [14]. Within the Australian health research environment, a historical emphasis on cultural awareness, which can ‘exoticize’ and ‘other’ First Nations peoples, rather than cultural proficiency, which seeks to encourage critical self-reflection and centre First Nations perspectives and cultures, has resulted in an evident failure of non-First Nations researchers to ethically contribute to First Nations health research, primarily through the devaluation of First Nations research methodologies within these settings [39,40].

While the concept of culturally proficient health research with First Nations communities remains a challenge for non-First Nations researchers, principles can provide guidance when considering research partnerships. These include philosophies such as community control over research activities (including what, when, where, and how research is to be undertaken), developing genuine and mutually beneficial research partnerships, facilitating reciprocal relationships to build the research capacity of both First Nations communities and non-First Nations researchers, allowing flexibility in research practice while maintaining scientific rigour, considering the impact of historical research practices within the local community, centering First Nations ways of knowing, being, and doing by considering, adopting, and applying Indigenist research methodologies as determined by the community, and demonstrating cultural humility [25,41,42,43,44,45,46,47]. Despite a raft of resources, as referred to, while constructive and empowering, are all but misguided without an adequate level of cultural proficiency of the non-First Nations research team. Researchers must be able to accurately interpret and apply the contextual-based information outlined in these resources while possessing a detailed understanding of the varying First Nations worldviews in a manner that creates positive research impacts for First Nations communities.

Maridulu Budyari Gumal is an academic health science partnership made up of 16 universities, hospitals, research institutes, community and primary care centres across the Sydney basin. The partnership has 16 Clinical Academic Groups (CAG) based on prioritised focus areas, including Aboriginal Health and Wellbeing, who have developed a set of introductory cultural proficiency resources targeted at researchers planning to research with Aboriginal communities in and around the Sydney basin. The online cultural proficiency resources available on the Maridulu Budyari Gumal website [48] include a video and checklist (Table 1) highlighting the researcher characteristics and steps required to build a culturally proficient research partnership with First Nations peoples and communities, along with a list of more detailed supplementary resources from various First Nations and non-First Nations research organisations. Further to these, the Aboriginal Health and Wellbeing CAG operates a 2 h Applied Cultural Proficiency for Researching in Indigenous Communities workshop, which non-First Nations researchers are encouraged to engage with and undertake regularly. The workshop explores various concepts and practical information including the impacts of colonisation on First Nations communities, trauma-informed research practice, Indigenist research methodologies, and culturally appropriate ways to establish a research agenda with First Nations communities.

This evaluation endeavoured to answer the following questions: Can the perceived cultural proficiency of non-First Nations researchers be developed through participation in a cultural proficiency workshop? Based on review of the online resources, do approaches to First Nations research engagement perceptions differ between First Nations and non-First Nations researchers? Furthermore, is it feasible to apply the information contained within the online resources to First Nations communities across Australia?

## 2. Materials and Methods

### 2.1. Materials

The resources were developed through First Nations community consultation within Sydney, Australia by the Aboriginal Health and Wellbeing CAG of Maridulu Budyari Gumal, also known as the Sydney Partnership for Health, Education, Research and Enterprise (SPHERE). The resources include a checklist (Table 1) for non-First Nations researchers to ensure they employ an appropriate and comprehensive approach to engagement and research with First Nations communities, as well as a video which raises important considerations when planning to engage with First Nations communities. The workshop is a 2 h interactive experience which explores several topics related to First Nations cultures, history, and research. Qualitative and quantitative instruments for the evaluation were developed by Maridulu Budyari Gumal’s Aboriginal Health and Wellbeing CAG.

### 2.2. Participants

Eligibility to participate in the evaluation was broad and included Australian-based health researchers (First Nations and non-First Nations). A purposive, convenience sampling technique was adopted. The evaluation involved three comparative groups to assess both disparities as well as similarities in findings. The comparative groups were determined to gain a deeper understanding of the two-eyed seeing model [49], theorising that an array of findings would indicate varying perspectives relating to appropriate research approaches with First Nations communities. The three groups comprised:

- Group 1—First Nations health researchers (evaluation of online resources only),
- Group 2—non-First Nations health researchers (evaluation of online resources only), and
- Group 3—non-First Nations health researchers (evaluation of online resources and pre- and post-workshop self-evaluation).

First Nations participants were included to ascertain: (1) the requirements of, and priorities for non-First Nations researchers when engaging with First Nations peoples and communities; and (2) the feasibility of broad application of the online resources across the various First Nations communities of Australia. Given the workshop was targeted at a local, Sydney basin level, and with local First Nations input into its design, evaluation by the Australia-wide First Nations participants was unjustified.

Non-First Nations participants were included to develop an understanding of: (1) the self-perceived efficacy of the cultural proficiency workshop; and (2) the priorities of non-First Nations researchers when engaging with First Nations peoples and communities. This cross-cultural approach to recruitment ensured a diversity in health research perspectives and experience of participants, with the authors anticipating significant variations in the research findings. 

### 2.3. Recruitment and Procedure

Non-First Nations researchers who participated in the evaluation of the online resources only were recruited via email invitation, with a link to the webpage where the resources are located. They were then provided with an online version of the evaluation form. Workshop participants were also sent an email inviting them to participate in the online resources’ evaluation prior to their involvement in the workshop. Invitation emails were distributed throughout the 16 partner organisations of Maridulu Budyari Gumal. Participants were provided with Participant Information Sheets via email and implied consent was sought through completion of the evaluation. Evaluations were anonymous.

First Nations participants were recruited during a First Nations health researchers conference in South Australia. The researchers introduced the research and provided a link where attendees could access the online resources. Attendees were then informed that physical Participant Information Sheets and evaluation forms would be available throughout the conference from the registration desk if interested in participating. The researchers returned at the end of the conference to obtain the completed evaluation forms. Implied consent was obtained via completion of the evaluation. Evaluations were anonymous.

### 2.4. Methods

Both qualitative and quantitative methods were adopted in a cross-cultural context This approach was employed to portray the complexities, necessity, and disparities of cultural proficiency when conducting research with First Nations communities. All participants were directed to the online video and checklist and asked to rate a series of statements. The online resource evaluation form was comprised of 2 sections: Quantitative statements utilising a 10-point Likert scale rating technique, where a score of 1 indicated ‘Strongly Disagree’ and a score of 10 indicated ‘Strongly Agree’; and a qualitative evaluation utilising a Strengths, Weaknesses, Opportunities, and Threats (SWOT) framework. A separate group of non-First Nations participants also evaluated the Applied Cultural Proficiency for Researching in Indigenous Communities workshop, providing pre- and post-workshop data pertaining to knowledge and skills acquisition adopting a similar 10-point Likert scale. All participant responses for the online resource evaluation were only identifiable through First Nations status. All responses attained from the workshop were anonymous.

### 2.5. Data Analysis

*T*-tests were conducted on quantitative data, including the calculation of *p*-values to assess statistical significance of the intervention for the non-First Nations group who undertook a pre and post workshop evaluation, as well as to assess any statistically significant variations between the First Nations and non-First Nations groups pertaining to the online resources’ evaluation. Thematic analysis [50] was applied to qualitative findings obtained via SWOT analysis to identify emerging themes. A manual approach to thematic analysis was employed.

## 3. Results

A total of 74 health researchers participated in the evaluation. 40 (54%) of these participants identified as First Nations. For the online resources’ evaluation, means were calculated across both non-First Nations groups (*n* = 34) and compared with the First Nations group (*n* = 40), respectively, to assess any variations in scores between the cultural groups (Table 2). Similarities in means were illustrated across the two cultural groups, with statistically insignificant findings for all statements. The mean for the non-First Nations group was compared to the First Nations group for statement 1, 8.225 (95% CI 7.79 to 8.66) and 8.25 (95% CI 7.79 to 8.71) *t*(58) = 0.123, *p* 0.45, respectively. For statement 2, the means were 6.525 (95% CI 6.07 to 6.99) and 6.333 (95% CI 5.65 to 7.01) *t*(77) = 0.45, *p* 0.33. Statement 3 means were calculated as 7.525 (95% CI 7 to 8.05) and 8 (95% CI 7.42 to 8.58) *t*(78) = −1.17, *p* 0.12. For statement 4, the averages were 7.2 (95% CI 6.54 to 7.86) and 7.5 (95% CI 6.84 to 8.16) *t*(78) = −0.62, *p* 0.27. Statement 5 averages were 8.85 (95% CI 8.46 to 9.24) and 8.325 (95% CI 7.84 to 8.81) *t*(78) = 1.63, *p* 0.054. For the final statement regarding missing information, the means for the two groups were calculated as 3.75 (95% CI 3.04 to 4.46) and 4.32 (95% CI 3.62 to 5.02) *t*(76) = −1.08, *p* 0.14.

Regarding the omission of key information in statement 6 of the online resource evaluation, most quantitatively disagreed that the resources had missed key information. Despite this, many provided comment to suggest that the resources had indeed missed key information. The dominating theme that emerged from the comments related to a lack of detailed information on ‘how’ to commence and maintain the engagement process with First Nations communities, within a research context. Many agreed that the resources were “a good starting point”, however stressed the need for more detailed information and complementary case studies to emphasise the nuanced information required from non-First Nations researchers when seeking to engage across different First Nations communities:

*“Be good to show actual process for a new researcher (but also applies to experienced researchers). Who is the group in your organisation that you need to talk to first and who will then introduce you to community elders (sic) within the specific community you will work with-obviously reciprocal-because if research is community driven-who do the elders approach in the research institute to help with the area of concern?”*

(non-First Nations participant)

As the resources were developed to be used in conjunction with key ethical guidelines, as mentioned within the online checklist, this finding suggests that perhaps those participants are unfamiliar with these ethical guideline resources [43,44,45,46] which provide more detailed information on practical approaches to research engagement with First Nations communities in Australia. It may also suggest that further resources are required, beyond ethical guideline documents, to develop the cross-cultural capability of non-First Nations researchers. This highlights the responsibility of non-First Nations research organisations and researchers to ensure their practices are informed by available resources and that they play an active role in understanding appropriate research practice with First Nations peoples and communities, rather than First Nations communities having the responsibility of guiding naïve researchers through these processes.

**Figure 1:**
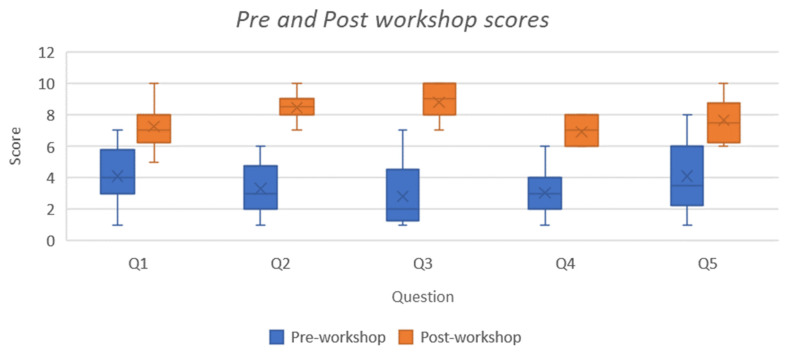
Pre- and Post-workshop Scores Distribution. Note: Pre and post workshop evaluation scores for the non-First Nations group.

Means were also calculated for the self-reported pre- and post-workshop evaluation (*n* = 20) (Table 3). For statement 1, the pre- and post-workshop mean was 4.1 (95% CI 3.33, 4.87) and 7.25 (95% CI 6.7, 7.8) *t*(38) = −6.35, *p* < 0.00001, respectively, indicating a significant increase in perceived ability. Statement 2, a pre and post mean of 3.3 (95% CI 2.62, 3.98) and 8.45 (95% CI 8.02, 8.88) *t*(38) = −12.25, *p* < 0.00001 was found, demonstrating a considerable increase in perceived knowledge. For statement 3, the means were 2.8 (95% CI 2.02, 3.58) and 8.8 (95% CI 8.39, 9.21) *t*(38) = −13.04, *p* < 0.00001, respectively, again showing a profound perceived impact of the intervention. In statement 4, a pre and post mean of 3.05 (95% CI 2.41, 3.69) and 6.9 (95% CI 6.51, 7.29) *t*(38) = −9.79, *p* < 0.00001 was calculated, demonstrating a significant change. The final statement produced an average pre- and post-workshop score of 4.1 (95% CI 3.15, 5.05) and 7.65 (95% CI 7.06, 8.24) *t*(38) = −6.06, *p* < 0.00001, respectively (see Figure 1).

For qualitative findings pertaining to the SWOT analysis, three (3) key themes emerged from the analysis of the Strengths section.

*Lived experience and diversity*

Many participants appreciated the ‘real-world experience’ that the video resource portrayed, this was related to the presence of experienced First Nations and non-First Nations health researchers in the video. Furthermore, participants found that the consideration of diversity within the video and online checklist, in respect to both the inclusion of First Nations and non-First Nations researchers in the video, as well as the need to consider the local context when attempting to undertake research with First Nations peoples and communities, was a strength of the resources:

“A mixture of Aboriginal and non-Aboriginal leaders within research and in the community sector. Listening to community/what do they need or want”

*Inclusion of important information*

Participants relayed that key information was included in the resources, such as the important role of Elders and community leaders in research engagement, community control of research, the significance of trust building between First Nations and non-First Nations stakeholders in research, and the value in aligning research goals with community expectations. These were again reported by both groups:

“Trust, taking time, listening, respecting leadership, having a shared idea. These are all important concepts that are clearly encouraged”

*Short, clear and engaging messaging*

Findings suggest that the resources offered short, sharp, and engaging content to consider when establishing a research partnership with First Nations communities. This was a common theme revealed in the analysis and was appreciated by those who had limited experience in First Nations-related research:

“Concise, clear, simple messages. Practiced advice on engagement for non-indigenous researchers especially”

A total of four (4) themes emerged following analysis of the Weaknesses section.

*None*

Many commented that the resources did not have any weaknesses, and this supports, to a degree, the quantitative results. These comments were prevalent within both the non-First Nations and First Nations groups, reflecting a variation in opinions relating to the considerations of researchers when engaging with First Nations peoples and communities.

*Impracticality of information*

A significant theme that the qualitative analysis revealed was a severe lack of practical knowledge pertaining to ‘how’ the information from the resources could be applied and implemented within a real-world setting. This theme was present in both groups:

“Doesn’t explain in depth how to translate the messages into practice”

“Maybe just a bit more detail on HOW to communicate effectively yet respectfully”

This was anticipated by the research team, who agree that the resources alone are not sufficient to ensure successful research partnership building with First Nations communities, given that they were designed to be used as introductory knowledge. Similar to the findings within the ‘missing key information’ section, this identified weakness from many participants reflects the need for non-First Nations researchers to play an active role in ensuring they know how to engage appropriately and respectfully with diverse First Nations peoples and communities within research contexts, initially through having a sound understanding of detailed guiding First Nations ethical research documents.

*Lack of community involvement*

A further theme related to the perceived lack of community involvement, despite the resources being dominated by First Nations voices and perspectives, including university staff, health service staff, and Aboriginal Community Controlled Health Organisation (ACCHO) staff. This may have included the absence of First Nations community members voices not attached to health and research organisations, which is a significant finding:

“Not really a weakness but could of (sic) involved community speaking in video”

“Probably needed some more community partners”

“More Aboriginal researcher voices needed (in the video)”

*Target audience not defined*

A further finding across both groups was the uncertainty related to who the resources were designed to target. While there was consensus within participants comments that non-First Nations researchers were the target audience for the resources, many questioned which First Nations communities would be the beneficiaries of the resources, including the relevance to Torres Strait Islander communities:

“I am still not sure who is the intended audience in which the information would ultimately benefit…”

“Not sure how it applies to Torres Strait Islander people”

Three (3) key themes resulted from the Opportunities component of the SWOT evaluation.

*Challenge beliefs*

Many participants believed that the resources would provide a platform to encourage conversations relating to appropriate ways of researching with First Nations peoples and communities, as well as contribute to a broader collective of First Nations-related research resources that challenge Western perspectives of research processes and approaches:

“To start a conversation about appropriate methodology for Aboriginal health research”

“I think that in the right setting, the video could garner conversations between Indigenous and non-Indigenous researchers”

*Consideration of alternative research methodologies*

Most participants across both groups commented on the resources providing an opportunity to consider alternative approaches to research, including the importance of First Nations research methodologies, such as, for example, the value of yarning as a research methodology:

“Rethink research approaches with First Nations communities”

“A new way to approach to conducting research with Aboriginal communities”

“To start a conversation about appropriate methodology for Aboriginal health research”

*Further educational opportunities*

Participants suggested the need for the availability of further, more detailed educational resources that could be linked with the current resources to expand their understanding in this field. Many commented on the need for follow-up resources that further explore this topic:

“A great base to build upon”

“Would be good as part of a broader package/education session on how to do good research with Aboriginal communities”

“Acts as a platform to launch more nuanced teaching materials”

Four (4) key Threats emerged from the analysis.

*None*

Similar to the weaknesses section, many participants within both the First Nations and non-First Nations groups commented that the resources harbored no particular threats, with many commenting that they didn’t perceive any potential threats associated with the resources.

*Interpreted as lip-service*

Participants within both groups felt that the information conveyed within the resources may be perceived as tokenistic and lack any real sincerity. This theme related to the ‘good intentions’ often expressed by non-First Nations people when seeking to conduct research with First Nations communities. This is an important finding as it relates to the notion of trust and a shared research agenda between First Nations peoples and non-First Nations researchers, something that has been an ongoing issue in First Nations-related research:

“…some may see it as a sugar-coated version of how Western epistemologies have drawn from Indigenous knowledge and strength without giving much back”

“It won’t move people beyond lip service”

*Underestimation of process*

Again, many participants from both groups noted that the engagement process portrayed within the resources was “easier said than done”, suggesting that the participants are aware of the complexities in establishing genuine research partnerships with First Nations communities. It was suggested that this is due to several factors including the biased history of First Nations-related research, the lack of community control over research and First Nations perspectives of Western research, and a dearth of collective understanding and skills of non-First Nations researchers working in First Nations-related research.

“Non-indigenous researchers might say “oh it’s too hard” and the community will miss out on important initiatives”

“Maybe simple to say but hard to do within the community. Every community is different and has different opinions of white research/ers”

“A researcher has a fixed idea, they may not like the idea of having to rethink their work”

“The resources are great but those not well versed in this area may see the suggestions as an easy fix”

*Elusiveness of tangible results*

A further finding was the perceived difficulty in attaining positive impact through a stepwise approach to establishing research partnerships:

“Perhaps a little formulaic—‘follow these steps and things will work.’ Also, might under-represent the actual difficulties in getting indigenous programs working successfully”

A significant theme that emerged overall from the analysis was a perceived lack of a pragmatic approach as to ‘how’ to apply the information appropriately and effectively in practice, despite direction to the accompanying detailed resources provided by First Nations and non-First Nations research organisations.

## 4. Discussion

While cultural proficiency was the model employed by the Maridulu Budyari Gumal resources, it is important to note that one can never be proficient in someone else’s culture, and this has been the main criticism of cultural competence and proficiency since its conception. The notion of always ‘becoming’, rather than ‘being’ culturally competent or proficient is a key conceptual philosophy adopted and argued by many cultural competence scholars [51,52,53]. While this notion may seem aspirational, it is important that non-First Nations researchers are aware of such an elusive goal, to recognise that their ability will always be imperfect. Practically, this is important due to the constant risk of failure in the engagement process, influenced by a range of personal and contextual factors, such as the range of approaches needed based on the community. While one can never be culturally proficient, the lessons learned in failure undoubtedly contribute to the development of cultural proficiency, and it is important that this notion does not deter researchers from pursuing their cultural proficiency journey. Contemporarily, the cultural safety model has been increasingly favoured by many First Nations peoples and communities due to its comprehensive approach when working cross-culturally with First Nations peoples and communities, including consideration and action on power differentials [28], however due to the identified resources and workshop applying a cultural proficiency lens, evaluation of such a model further contributes to the evidence-base in this field.

The overall statistically insignificant comparison made between the two cultural groups following the resource examination suggests a similar level of understanding and agreement in terms of resource acceptability and need. Relatively comparable findings also regarding qualitative responses illustrates the critical reflection that participants expressed while completing the evaluation. Given the significant experience of the First Nations cohort in Indigenist research methodologies and research conduct with First Nations communities, the general similarity in findings suggests a great deal of understanding of the non-First Nations cohort regarding the considerations to research engagement with First Nations peoples and communities. There was a statistically significant difference in pre- and post-workshop scores across all questions when analysed, indicating the self-perceived effectiveness of the workshop for non-First Nations researchers. While both the online resources and workshop enhanced the perceived understanding of non-First Nations participants, it is difficult to measure the true extent of the change in applied cultural proficiency through a self-reporting approach. Ideally, evaluation of non-First Nations participants by First Nations peoples and communities would more accurately assess the practical impact of the interventions on non-First Nations researchers.

The non-First Nations group were less likely to agree that the material could benefit both First Nations and non-First Nations researchers, implying a belief that perhaps First Nations researchers do not require cultural proficiency training. This notion is contentious, as First Nations peoples and communities are not homogenous, and can differ quite significantly in terms of research engagement perspectives and protocols. Furthermore, there are many First Nations people who have only recently been made aware of their Indigeneity or who have been raised in a Westernised setting with limited experience in First Nations cultures. This supports the uninformed stereotype that First Nations people know everything about all First Nations cultures within Australia, which is a significantly flawed assumption, and can be problematic when non-First Nations researchers are ignorant of this and encourage First Nations people to engage with communities with which they are not previously connected, to facilitate the research engagement process [54].

When compared to the First Nations group, the non-First Nations cohort were also more likely to believe that the resources could not be applied broadly across all First Nations communities. This outcome demonstrates great insight into the diversity of community protocols related to engagement and research between different First Nations communities throughout Australia. While the idea of broad application was more favourable among the First Nations cohort, due to the introductory nature of the resources, the notion was highly supported by the non-First Nations group also, inferring applicability of the underlying resource philosophies to multiple, diverse First Nations communities. This finding is supportive of the literature [42] and speaks to the overarching similarities in values inherent within Australian First Nations populations, such as respect, reciprocity, responsibility, community-control, and the importance of connection to culture (Kin, Land, and Lore). Furthermore, the non-First Nations group were more likely to agree that the resources should be a part of a larger training package, suggesting a need for research institutions to provide evidence-based, consistent, ongoing, and thorough cultural proficiency education opportunities to all research staff, irrespective of whether they conduct research with First Nations populations. This belief was also strongly supported by the First Nations cohort, further indicating the necessity for a comprehensive approach to cultural proficiency development among health researchers.

A striking finding of the research was an emphasis on a lack of direction related to ‘how’ the information from the resources could or should be implemented within a real-world context, despite the positive self-reported results attained from the workshop evaluation. Many participants agreed that while the resources provided a good starting point for novice researchers to be informed in this space, there was much uncertainty regarding the application of the knowledge to affect positive outcomes in research engagement with First Nations peoples and communities. This finding highlights the disparity between the perceived understanding of non-First Nations researchers and their ability to apply the knowledge within practice, as well as a broader lack of experience related to research engagement with First Nations peoples and communities. While resources and workshops can guide researchers when engaging with First Nations communities, the limitation in passive cross-cultural learning is clear [55]. The application of cultural proficiency learnings requires researchers to appreciate key concepts, such as the importance of centring Indigenist research methodologies to attain self-determination for First Nations peoples, the importance of community-control over research, and a detailed understanding of the role of allies in First Nations research spaces [56] p. 320. Furthermore, non-First Nations researchers’ engagement and research approaches must be underpinned by cultural humility [31] to ensure they are accountable for their practices. Beyond these considerations, non-First Nations researchers must value and respect local community protocols related to engagement and research. This can vary immensely and often commences with an informal interaction, a practice Bessarab and Ng’andu refer to as ‘social yarning’ [57]. Social yarning involves the informal introduction of two or more parties to ‘get to know’ each other and understand who and where people come from. This approach is often commonplace in many First Nations cultures and commences a process of rapport building that establishes contextual accountability and responsibility, a concept that is vital in relationships among many First Nations peoples and communities. It is the responsibility of non-First Nations research institutes, researchers, and funding bodies to understand and ensure that:

1. Researchers must demonstrate cultural proficiency in the context of First Nations research (i.e., critical self-reflection, cultural humility, respect and adhere to local community protocols, be aware that you are a guest within the community, community-control over research).
2. Researchers recognise the importance of ongoing, respectful, and reciprocal relationships and engagement for First Nations peoples and communities, with research relationships no exception.
3. Processes, activities, and timelines related to research with First Nations peoples and communities align with the expectations and local protocols of these communities, for example, extending research funding to allow for a potentially long engagement process to build trust prior to any research being undertaken.
4. Research is continuously directed by First Nations peoples and communities, including what, where, when, and how research is undertaken.
5. Engagement attempts and the perceived success of these attempts by non-First Nations researchers does not always translate to community acceptance of any future research activities. This may be due to the research proposal not aligning to the needs of the community, unfavourable past experiences with research, or competing community priorities, such as Sorry Business, to name a few.
6. Research institutions consider the varying degrees of cross-cultural capability of their researchers, and personalized education and training opportunities are available to ensure consistently positive outcomes in research-related encounters with First Nations communities.

The reported discord between perceived ability and capacity to implement the knowledge suggests that the introductory resources alone are not sufficient to develop cultural proficiency, as anticipated by the authors, and expressed within the literature. However, the insight from the non-First Nations participants, in terms of recognising this inadequacy, provides some hope regarding the perceived understanding of the commitment required to develop culturally proficient research partnerships with First Nations peoples and communities.

The self-reported, statistically significant effect of the workshop intervention indicates that cultural proficiency education and training should involve a considerable interactive component where participants are able to immerse themselves within First Nations epistemologies, ontologies, and axiologies, and critically analyse their beliefs, assumptions, and values. This should ideally be facilitated by local First Nations peoples with significant knowledge of Australian First Nations cultures and history, as well as Indigenist research methodologies. The literature supports interactive training regarding cross-cultural capability development in the same way that hands-on learning has been proven effective within the education setting [58].

Cultural proficiency workshops and training opportunities should inherently incite critical self-reflection through challenging discussions regarding former events and policies that have influenced the inequitable outcomes seen in First Nations research [59]. Self-reflection leading to self-reflexivity should be the aim of any cultural proficiency training program, creating the ability to consciously alter attitudes and behaviours based on an accurate understanding of self-beliefs and the beliefs of others, particularly when faced with an unfavourable outcome. The ability to be self-reflexive ensures that any mistakes made, are critically examined by the researcher to improve future approaches to engagement. This should continue indefinitely with critical self-reflection, a constant process throughout the life-long journey of cross-cultural capability development. A contextually driven approach to cross-cultural capability as it relates to First Nations research has been established both within the literature as well as from the resulting evaluation (p. 70, [46,60]). The bespoke disposition encourages flexibility based on the local First Nations culture and goes far to disregard a global approach to cultural proficiency education and training. Despite this, the higher-level principles evident within many ethical documents should be applied to underpin any cross-cultural training pertinent to First Nations research. These principles guide a respectful approach that encourages power-balance, self-determination, and social inclusion more broadly, facilitating a decolonising partnership. Employing these principles in a way that respects local protocols and gives voice to the community as directors of First Nations research demonstrates culturally proficient research practice.

### Limitations

Several limitations were identified in the evaluation of the online resources as well as the cultural proficiency workshop. While a reliable tool for resource evaluation, a cross-sectional survey captures only glimpses of information without necessarily exploring perspectives in detail. This may have impacted on analysis and interpretation of the data and findings. Further to this, differing recruitment and procedural approaches may have impacted on the consistency of participants understanding in relation to both the resources and the evaluation. This is unlikely due to the clear explanation that accompanied the invitation to participate in the evaluation. While convenience sampling was used for the research, potentially contributing a degree of inherent bias to the evaluation, it is important to note that a service directory of Maridulu Budyari Gumal was utilised to source many of the non-First Nations participants, highlighting participant affiliation with the network that developed the resource.

Another limitation identified in the evaluation was the relatively small sample with which the research was conducted, as well as a lack of a control group to compare with the workshop cohort. As the resource evaluation tool was different to the workshop evaluation, there was no control group to assess the impact of the workshop intervention. However, this limitation was addressed by the completion of a pre-workshop evaluation, providing a self-reported baseline assessment which could then be compared with the post-workshop results.

The authors note that self-evaluation of cross-cultural capability allows for varying degrees of perceived ability and optimism in non-First Nations researchers, and this is a further limitation of this research. The evaluation identified several shortcomings pertaining to the cultural competency/proficiency model, including the notion of ‘perceived’ vs. ‘actual’ cross-cultural capability of non-First Nations researchers through self-evaluation, an issue that cultural safety considers through community appraisal. A further limitation identified was the issue of implementation of the cultural proficiency education and training, highlighted by participants uncertainty in ‘how’ to action the perceived knowledge and skills attained through the resources. This may have been due to the function of the resources as introductory materials for novice researchers; however, it does focus attention on the practical capacity of the resources, beyond perceived capability.

## 5. Conclusions

The online resources and workshop developed by Maridulu Budyari Gumal are an example of a collaborative cultural proficiency education and training resource developed by First Nations health and research staff, and local First Nations community members, that encourages an inclusive, self-determining approach to First Nations health research. A focus on proficiency, rather than awareness demonstrates the understanding and commitment required from non-First Nations researchers to engage appropriately and effectively with First Nations peoples and communities. While the online resources alone did not adequately contribute to the perceived development of cultural proficiency across the participant groups, those non-First Nations participants who additionally attended a cultural proficiency workshop did indicate an increase in self-reported cultural proficiency, although this was unsubstantiated within a First Nations engagement context. Results from the evaluation support a holistic, ongoing approach of informative material, practical recommendations, and immersion in local First Nations community knowledges and practices.

Regarding the scope of application of the resources, the evaluation indicated that while context is important in the development of cultural proficiency education and training material, broad application of the online resources throughout Australia, to inform non-First Nations researchers of the importance of Indigenist research methodologies, as well as guide First Nations engagement considerations for non-First Nations researchers is supported. This outcome was primarily due to the overarching principles evident throughout the material, highlighting values such as respect, integrity, beneficence, reciprocity, responsibility, and self-determination. Non-First Nations researchers must be able to implement the knowledges and skills attained within cultural proficiency education and training, ideally validated by local First Nations peoples and communities in which research is to be planned and undertaken.

Further practical evaluation of the ‘perceived’ cultural proficiency of participants is needed to establish the true efficacy of the workshop. Moreover, a consistent, objective, and validated process for measuring cultural proficiency within a First Nations research context is required. This will eliminate the ‘perceived’ element of cultural proficiency and contribute to improving outcomes within cross-cultural research partnerships with First Nations communities.

## Figures and Tables

**Table 1:** Maridulu Budyari Gumal’s Aboriginal Health and Wellbeing Clinical Academic Group ‘Steps to success’ checklist.

No.	Step	Consideration
1	Confirm your core values fit with Indigenist research	Complete Maridulu Budyari Gumal website’s on-line Applied Cultural Proficiency for Researching in Indigenous Communities learning module, and/or the cultural competence education at your organisation
2	Embed a capacity building methodology	Use of an appropriate methodology such as Community Based Participatory Action Research; Dadirri; the Dilly Bag Model; or the model best suited to your community research
3	Know your country and community leaders	Letter of support from relevant community organisation and/or Elders
4	Be ethics-minded	Ethics application supported by your organisation, the Aboriginal Health and Medical Research Council; your host Aboriginal community organisation/s
5	Collaborate with appropriate research bodies within your organisation	Cultural endorsement of your project (for example, an Elders or Community Committee, and/or Community Boards in the host Aboriginal Organisations).
6	Ensure your project demonstrates research reciprocity	Intellectual Property rights are agreed on and shared across research partners as appropriate. Measurable long-term benefits to community (as determined by the community).
7	Evaluate and translate your research	Evaluation of the research and outcomes from an Indigenist lens (for example, using the Ngaa-bi-nya Framework for Aboriginal and Torres Strait Islander program evaluation). Research is translated into outcomes and outputs useful for researchers, policy makers, health practitioners, and your host community.
8	Support your research to be an agent of change in Aboriginal communities	Demonstrate community ownership of research project and process. Demonstrate capacity building within Aboriginal communities (for example, by the inclusion of Aboriginal Researchers in research leadership positions).

**Table 2:** Online Resource Evaluation Results (video and checklist).

No.	Evaluation Statements	Mean (Non-First Nations	Mean (First Nations)	t-Value	*p*-Value
1	The resources clearly outlined what is needed to create an effective partnership with First Nations communities	8.225	8.25	0.123	0.45
2	I am more confident in conducting research with First Nations communities after viewing the resources	6.525	6.333	0.45	0.33
3	The resources can benefit both First Nations and non-First Nations researchers	7.525	8	−1.17	0.12
4	The resources can be applied universally to create an effective partnership with all First Nations communities in Australia	7.2	7.5	−0.62	0.27
5	The resources should be included as part of a larger cultural proficiency training package available to all researchers conducting or planning to conduct research with First Nations communities	8.85	8.325	1.63	0.054
6	The resources missed key information Please explain:_________________________	3.75	4.32	−1.08	0.14

**Table 3:** Pre- and Post-Workshop Evaluation Results.

No.	Evaluation Statements	Mean (Pre-Workshop)	Mean (Post-Workshop)	t-Value	*p*-Value
1	I can discuss the history of First Nations people	4.1	7.25	−6.35	<0.00001
2	I understand the different values in First Nations research	3.3	8.45	−12.25	<0.00001
3	I am able to discuss a model of Indigenist research	2.8	8.8	−13.04	<0.00001
4	I can apply an Indigenist model of evaluation to research	3.05	6.9	−9.79	<0.00001
5	I am confident in engaging with First Nations communities	4.1	7.65	−6.06	<0.00001

## Data Availability

The data generated and analysed during the study are not publicly available because study participants did not consent to the data being made available to other researchers.
